# Acute respiratory distress syndrome is associated with impaired alveolar macrophage efferocytosis

**DOI:** 10.1183/13993003.00829-2021

**Published:** 2021-09-09

**Authors:** Rahul Y. Mahida, Aaron Scott, Dhruv Parekh, Sebastian T. Lugg, Rowan S. Hardy, Gareth G. Lavery, Michael A. Matthay, Babu Naidu, Gavin D. Perkins, David R. Thickett

**Affiliations:** 1 Birmingham Acute Care Research Group, Institute of Inflammation and Ageing, University of Birmingham, Birmingham, UK; 2 Institute of Metabolism and Systems Research, University of Birmingham, Birmingham, UK; 3 Dept of Medicine, and Dept of Anaesthesia, Cardiovascular Research Institute, University of California San Francisco, San Francisco, CA, USA; 4 Emergency, Pre-hospital, Perioperative and Critical Care Group, Warwick Medical School, University of Warwick, Warwick, UK; 5 Joint first authors; 6 Joint senior authors

## Abstract

Acute respiratory distress syndrome (ARDS) is an inflammatory disorder of the lungs, with sepsis as the predominant aetiology. Despite advances in ventilation strategies, mortality for moderate to severe ARDS remains at 40–46% [1]. ARDS is associated with neutrophil influx into alveoli. Persistently high neutrophil and low alveolar macrophage (AM) numbers in bronchoalveolar lavage (BAL) fluid are associated with greater mortality [2]. While the inflammatory alveolar environment of early ARDS initially delays apoptosis, these neutrophils ultimately undergo apoptosis within alveoli [3]. Efficient efferocytosis of apoptotic neutrophils by AMs is critical for resolution of inflammation [3]. Apoptotic neutrophils may accumulate in ARDS due to defective AM efferocytosis and/or overwhelmed efferocytosis capacity, then undergo secondary necrosis, releasing inflammatory mediators into the alveolar space [4]. This may contribute to the prolonged inflammation observed in ARDS. No study has previously assessed AM efferocytosis in ARDS; however, monocyte-derived macrophages (MDMs) from ARDS patients do have impaired efferocytosis [5]. We investigated whether ARDS patients have impaired AM efferocytosis and increased alveolar neutrophil apoptosis.

## Abstract 2

**ARDS patients have decreased alveolar macrophage efferocytosis, which is associated with increased alveolar inflammation, and may contribute to worse clinical outcomes, including mortality. Upregulation of efferocytosis may offer a therapeutic strategy.** https://bit.ly/2Q7REdM

*To the Editor*:

Acute respiratory distress syndrome (ARDS) is an inflammatory disorder of the lungs, with sepsis as the predominant aetiology. Despite advances in ventilation strategies, mortality for moderate to severe ARDS remains at 40–46% [1]. ARDS is associated with neutrophil influx into alveoli. Persistently high neutrophil and low alveolar macrophage (AM) numbers in bronchoalveolar lavage (BAL) fluid are associated with greater mortality [2]. While the inflammatory alveolar environment of early ARDS initially delays apoptosis, these neutrophils ultimately undergo apoptosis within alveoli [3]. Efficient efferocytosis of apoptotic neutrophils by AMs is critical for resolution of inflammation [3]. Apoptotic neutrophils may accumulate in ARDS due to defective AM efferocytosis and/or overwhelmed efferocytosis capacity, then undergo secondary necrosis, releasing inflammatory mediators into the alveolar space [4]. This may contribute to the prolonged inflammation observed in ARDS. No study has previously assessed AM efferocytosis in ARDS; however, monocyte-derived macrophages (MDMs) from ARDS patients do have impaired efferocytosis [5]. We investigated whether ARDS patients have impaired AM efferocytosis and increased alveolar neutrophil apoptosis.

Ethical approval was obtained to recruit ventilated sepsis patients with and without ARDS (REC 16/WA/0169). For patients without capacity, permission to enrol was sought from a legal representative. Invasively ventilated adult patients with sepsis were recruited from the intensive care unit (ICU) of Queen Elizabeth Hospital Birmingham, UK from 2016–2019. Sepsis was defined according to Sepsis-3 criteria [6]. Patients who fulfilled the Berlin criteria [7] within the previous 48 h were classified as having ARDS, whereas those without ARDS (lacking bilateral infiltrates) were defined as controls. Exclusion criteria included imminent treatment withdrawal, steroid therapy prior to admission, abnormal clotting precluding bronchoscopy, and clinically relevant immunosuppression. Patients underwent bronchoscopy with BAL within 48 h of initiation of invasive ventilation.

AMs were isolated from patient BAL using Lymphoprep (StemCell™) density gradient centrifugation and plastic adherence [8]. After 24 h, AMs were used in flow cytometric efferocytosis assays with CellTracker™ Deep Red (ThermoFisher) labelled heterologous neutrophils [9]. BAL neutrophil apoptosis and necrosis were assessed by flow cytometric Annexin V Apoptosis Detection Kit (BioLegend) and cytospin morphology. BAL cytokines were quantified by Luminex assays (R&D Systems).

Of the 38 ventilated sepsis patients recruited, 21 had ARDS, the remainder being controls. Of the control patients, four developed ARDS later in their admission. There was no significant difference in age (mean±sd 59.2±13.9 *versus* 55.1±16.3 years; p=0.42), sex (71% *versus* 65% male; p=0.73), or smoking status between sepsis patients with and without ARDS. The ratio of arterial partial pressure of oxygen to fraction of inspired oxygen (P/F ratio) on admission to ICU was also not significantly different between patient groups (21.8±4.9 *versus* 24.1±6.8; p=0.27). At ICU admission, one (4.8%) of the ARDS patients had mild severity, 18 (85.7%) had moderate severity and two (9.5%) had severe ARDS as per Berlin criteria [7]. Pneumonia was the predominant source of sepsis in both groups (90% *versus* 71%; p=0.21). Ventilator settings including positive end-expiratory pressure, driving pressure, plateau pressure, and tidal volume were not significantly different between patient groups. Sequential Organ Failure Assessment (SOFA; 12.5±3.8 *versus* 10.3±2.7; p=0.053) and Acute Physiology And Chronic Health Evaluation-2 (APACHE II: 18.6±5.5 *versus* 15.2±5.8; p=0.091) severity scores were not significantly different between groups. However, the Murray Lung Injury score was greater in sepsis patients with ARDS (2.57±0.5 *versus* 2.13±0.46; p=0.009). Further physiological parameters are reported in our associated preprint [10]. Regarding outcomes, inpatient mortality was seven (33.3%) in sepsis patients with ARDS and three (17.6%) in those without ARDS. Ventilator-free days to day 28 were lower in patients with ARDS (6.9±9.2 *versus* 15.9±8.3 days; p=0.004) and ICU length of stay was longer (median (interquartile range) 23.0 (12.8–33.8) *versus* 12.0 (7.5–19.0) days; p=0.004).

BAL was collected from 31 patients (17 ARDS, 14 control). BAL total leukocyte count was not significantly different between patient groups (median 15.8 (7.4–31.3) ×10^6^ *versus* 6.4 (3.8–27.0) ×10^6^; p=0.133). However, BAL neutrophil count was higher in patients with ARDS (median 14.8 (5.4–27.8) ×10^6^ *versus* 3.2 (1.0–8.7) ×10^6^; p=0.023). The proportion of patients with positive BAL cultures on microbiological analysis was not significantly different between groups (76.5% *versus* 78.6%; p>0.99). BAL neutrophil apoptosis was assessed in 21 patients (12 ARDS, nine control). Neutrophil apoptosis and necrosis were not initially assessed and were added to the study protocol after recruitment had begun. BAL AM yield (mean 1.2 million) was sufficient to perform efferocytosis in 22 patients (11 ARDS, 11 control).

AM efferocytosis was impaired in sepsis patients with ARDS compared to those without ARDS (figure 1a; 7.6% *versus* 22.7%; p=0.003). Alveolar neutrophil apoptosis (assessed immediately post-BAL) was greater in sepsis patients with ARDS compared to those without ARDS (figure 1b; 41.3% *versus* 14.1%; p=0.0001). Across all sepsis patients (with and without ARDS), a trend towards negative correlation between AM efferocytosis and BAL neutrophil apoptosis was observed, but this did not reach statistical significance (figure 1c; r=−0.525, p=0.057). A trend towards increased alveolar neutrophil necrosis in sepsis patients with ARDS compared to those without ARDS was observed, but this did not reach statistical significance (figure 1d; medians 4.5% *versus* 1.1%; p=0.162). Across all sepsis patients (with and without ARDS), AM efferocytosis showed negative correlation with BAL concentrations of IL-8 (figure 1e; r=−0.707, p=0.0003), and IL-1ra (figure 1f; r=−0.601, p=0.004). For all sepsis patients (with and without ARDS), low AM efferocytosis index was associated with reduced freedom from mechanical ventilation (figure 1g; p=0.015) and increased mortality in the 30 days from enrolment (figure 1h; p=0.013).

**FIGURE 1:**
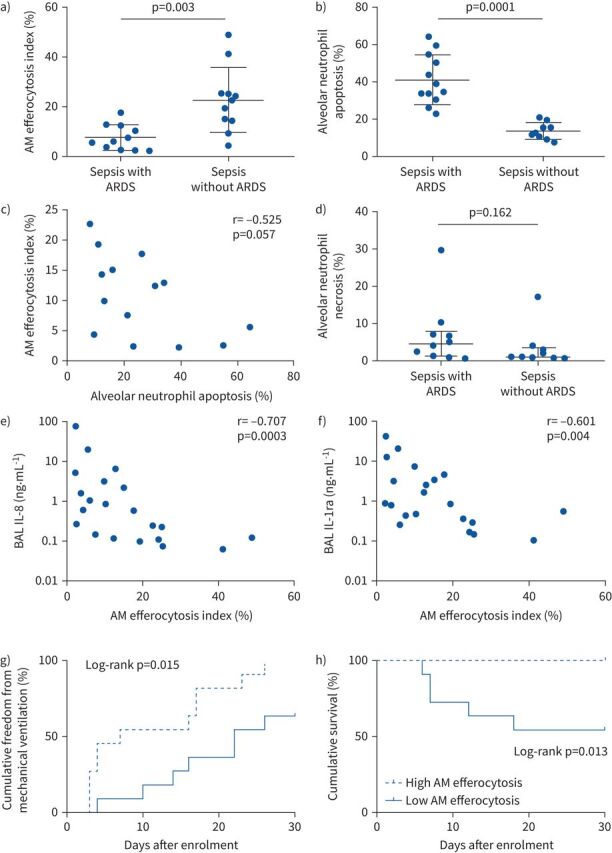
a) Sepsis patients with acute respiratory distress syndrome (ARDS) have significantly reduced alveolar macrophage (AM) efferocytosis index compared to sepsis patients without ARDS (means 7.6 *versus* 22.7%; p=0.003). Error bars shown as mean±sd. Statistical analysis by Welch's t-test, n=11–12 in all groups. b) Neutrophil apoptosis assessed within 1 h of bronchoalveolar lavage (BAL) fluid collection. Sepsis patients with ARDS have significantly greater percentage of apoptotic neutrophils in BAL compared to sepsis patients without ARDS (means 41.3 *versus* 14.1%; p=0.0001). Error bars shown as mean±sd. Statistical analysis by Welch's t-test, n≥9 in both groups. Some patients did not have neutrophil apoptosis and necrosis measured since this was only added to the study protocol after recruitment had already begun. c) AM efferocytosis index *versus* BAL neutrophil apoptosis in all sepsis patients (with and without ARDS). A trend towards negative correlation was observed between AM efferocytosis and BAL neutrophil apoptosis, but this did not reach statistical significance (n=14; r=−0.525, p=0.057). Statistical analysis by Spearman's correlation coefficient. d) Neutrophil necrosis assessed within 1 h of BAL fluid collection. No significant difference in BAL neutrophil necrosis was observed between sepsis patients with or without ARDS (medians 4.5 *versus* 1.1%; p=0.162). Error bars shown as median (interquartile range). Statistical analysis by Mann–Whitney U-test, n≥9 in both groups. e and f) Levels of interleukin 1 receptor antagonist (IL-1ra) and IL-8 were measured in BAL from sepsis patients with and without ARDS, then correlated with AM efferocytosis index. There is significant negative correlation between AM efferocytosis index and BAL concentrations of IL-8 (r=−0.707, p=0.0003) and IL-1ra (r=−0.601, p=0.004) in sepsis patients with and without ARDS. Log scales used for both graphs, semi-log non-linear line of fit used, n=21 for both plots. Spearman's correlation coefficient with Bonferroni's correction used for statistical analysis. Bonferroni corrected significance p<0.00625. g) A threshold AM efferocytosis index of 12.7% was used to distinguish between “low” and “high” efferocytosis, based on this value being 1 standard deviation above the mean AM efferocytosis index of sepsis patients with ARDS. For all sepsis patients (with and without ARDS), low AM efferocytosis index was associated with reduced freedom from mechanical ventilation in the 30 days from enrolment (chi-squared 7.41, p=0.015). Statistical analysis by log-rank test, n=11 in each group. h) For all sepsis patients (with and without ARDS), low AM efferocytosis index was associated with decreased survival in the 30 days from enrolment (chi-squared 6.22, p=0.013). Statistical analysis by log-rank test, n=11 in each group.

A previous study [5] showed that MDMs from ARDS patients had impaired efferocytosis. Our study identified an impairment in AM efferocytosis, which supports these findings and is more relevant to ARDS pathogenesis, since the disease process originates in the alveoli. Our control group consisted of ventilated sepsis patients, in contrast to this previous study, which included patients undergoing outpatient bronchoscopy as controls [5]. Therefore, our study determined that the decrease in AM efferocytosis associated with the development of sepsis-related ARDS occurs independently of sepsis, ICU admission and invasive ventilation.

We postulated that decreased AM efferocytosis in ARDS may be due to polarisation of AMs to a pro-inflammatory phenotype, which is associated with reduced efferocytosis [11]. Further studies are required to investigate this potential association. Negative correlations were observed between AM efferocytosis and BAL cytokines IL-8 and IL-1ra. IL-8 induces classical activation of macrophages, which is associated with decreased efferocytosis. Blockade of IL-8 may be a potential strategy to upregulate efferocytosis, attenuate inflammation, and reduce duration of mechanical ventilation. Medications known to enhance efferocytosis (*e.g.* glucocorticoids) could be tested in ARDS models [12].

The accumulation of apoptotic alveolar neutrophils observed in ARDS patients could be due to increased apoptosis and/or decreased clearance. Previous studies showed that alveolar and circulating neutrophils from ARDS patients showed delayed apoptosis following *ex vivo* culture for 20 h [13]. However, these results cannot be directly compared with those from our study, in which alveolar neutrophil apoptosis was assessed immediately after BAL (without *ex vivo* culture). Only two studies have previously investigated alveolar neutrophil apoptosis in early ARDS immediately post-BAL [14, 15]. Neither study showed a difference in alveolar neutrophil apoptosis between ARDS and control patients; furthermore, the trends observed were contradictory. Therefore, only limited comparisons can be made with our study. Our data suggest that alveolar neutrophil apoptosis is initially delayed due to the pro-inflammatory contents of ARDS BAL, but once apoptosis occurs, the neutrophils persist due to impaired AM efferocytosis.

Our study had limitations; ARDS patient recruitment was limited because prior steroid therapy and immunosuppression were exclusion factors. Some patients could not safely undergo bronchoscopy due to their ventilation status; no patients with severe ARDS underwent bronchoscopy for this reason. BAL from ARDS patients was highly neutrophilic, making AM isolation difficult. Mean BAL AM yield was 1.2 million, meaning that often only one functional assay could be performed per patient; efferocytosis was given priority. Efferocytosis assays were undertaken with heterologous (not autologous) neutrophils *ex vivo*.

In summary, our findings indicate that patients with sepsis-related ARDS have impaired AM efferocytosis, which potentially contributes to ARDS pathogenesis and negatively impacts clinical outcomes, including mortality. Strategies to upregulate AM efferocytosis may be of value for attenuating inflammation in ARDS.

## Shareable PDF

10.1183/13993003.00829-2021.Shareable1 This one-page PDF can be shared freely online.
Shareable PDF ERJ-00829-2021.Shareable
