# Supplementary material for: Acute respiratory distress syndrome is associated with impaired alveolar macrophage efferocytosis
Source: Eur Respir J. 2021 Sep 9;58(3):2100829. doi: 10.1183/13993003.00829-2021 (PMC8754102; doi:10.1183/13993003.00829-2021)
Supplement: Supplementary file 1 [file ERJ-00829-2021.Shareable.pdf]

SHAREABLE PDF

Acute respiratory distress syndrome is associated with impaired alveolar macrophage efferocytosis

Rahul Y. Mahida [1,5], Aaron Scott [1,5], Dhruv Parekh [1], Sebastian T. Lugg [1], Rowan S. Hardy [2], Gareth G. Lavery [2], Michael A. Matthay [3], Babu Naidu [1], Gavin D. Perkins [4,6] and David R. Thickett [1,6]

[1]Birmingham Acute Care Research Group, Institute of Inflammation and Ageing, University of Birmingham, Birmingham, UK. [2]Institute of Metabolism and Systems Research, University of Birmingham, Birmingham, UK. [3]Dept of Medicine, and Dept of Anaesthesia, Cardiovascular Research Institute, University of California San Francisco, San Francisco, CA, USA. [4]Emergency, Pre-hospital, Perioperative and Critical Care Group, Warwick Medical School, University of Warwick, Warwick, UK. [5]Joint first authors. [6]Joint senior authors.

Rahul Y. Mahida (r.mahida@bham.ac.uk)

Check for updates

Shareable abstract (@ERSpublications)
ARDS patients have decreased alveolar macrophage efferocytosis, which is associated with increased alveolar inflammation, and may contribute to worse clinical outcomes, including mortality. Upregulation of efferocytosis may offer a therapeutic strategy. https://bit.ly/2Q7REdM

Cite this article as: Mahida RY, Scott A, Parekh D, *et al*. Acute respiratory distress syndrome is associated with impaired alveolar macrophage efferocytosis. *Eur Respir J* 2021; 58: 2100829 [DOI: 10.1183/13993003.00829-2021].

This single-page version can be shared freely online.

Copyright ©The authors 2021.

This version is distributed under the terms of the Creative Commons Attribution Licence 4.0.

Received: 12 Feb 2021
Accepted: 6 May 2021

To the Editor:

Acute respiratory distress syndrome (ARDS) is an inflammatory disorder of the lungs, with sepsis as the predominant aetiology. Despite advances in ventilation strategies, mortality for moderate to severe ARDS remains at 40–46% [1]. ARDS is associated with neutrophil influx into alveoli. Persistently high neutrophil and low alveolar macrophage (AM) numbers in bronchoalveolar lavage (BAL) fluid are associated with greater mortality [2]. While the inflammatory alveolar environment of early ARDS initially delays apoptosis, these neutrophils ultimately undergo apoptosis within alveoli [3]. Efficient efferocytosis of apoptotic neutrophils by AMs is critical for resolution of inflammation [3]. Apoptotic neutrophils may accumulate in ARDS due to defective AM efferocytosis and/or overwhelmed efferocytosis capacity, then undergo secondary necrosis, releasing inflammatory mediators into the alveolar space [4]. This may contribute to the prolonged inflammation observed in ARDS. No study has previously assessed AM efferocytosis in ARDS; however, monocyte-derived macrophages (MDMs) from ARDS patients do have impaired efferocytosis [5]. We investigated whether ARDS patients have impaired AM efferocytosis and increased alveolar neutrophil apoptosis.
